# Co-expression network analysis identifies Spleen Tyrosine Kinase (SYK) as a candidate oncogenic driver in a subset of small-cell lung cancer

**DOI:** 10.1186/1752-0509-7-S5-S1

**Published:** 2013-12-09

**Authors:** Akshata R Udyavar, Megan D Hoeksema, Jonathan E Clark, Yong Zou, Zuojian Tang, Zhiguo Li, Ming Li, Heidi Chen, Alexander Statnikov, Yu Shyr, Daniel C Liebler, John Field, Rosana Eisenberg, Lourdes Estrada, Pierre P Massion, Vito Quaranta

**Affiliations:** 1 Department of Cancer Biology, Vanderbilt University, 2220 Pierce Avenue, Nashville, TN 37232, USA; 2 Department of Biochemistry, Vanderbilt University, 2220 Pierce Avenue, Nashville, TN 37232, USA; 3 Department of Biostatistics, Vanderbilt University, 2220 Pierce Avenue, Nashville, TN 37232, USA; 4 Department of Pharmacology, Vanderbilt University, 2220 Pierce Avenue, Nashville, TN 37232, USA; 5 Department of Pathology, Vanderbilt University, 2220 Pierce Avenue, Nashville, TN 37232, USA; 6 Divisions of Cancer Biostatistics, Vanderbilt University, 2220 Pierce Avenue, Nashville, TN 37232, USA; 7 Allergy/Pulmonary & Critical Care Medicine, Vanderbilt University, 2220 Pierce Avenue, Nashville, TN 37232, USA; 8 Center for Cancer Systems Biology, Vanderbilt University, 2220 Pierce Avenue, Nashville, TN 37232, USA; 9 Veterans Affairs, Tennessee Valley Healthcare System, Nashville Campus, TN 37232, USA; 10 Center for Health Informatics and Bioinformatics, New York University Langone Medical Center, New York, NY 10016, USA; 11 Department of Medicine, New York University School of Medicine, New York, NY 10016, USA; 12 Department of Molecular and Clinical Cancer Medicine, University of Liverpool, Liverpool, Merseyside L69 3BX, UK

**Keywords:** Co-expression network, Small-cell lung cancer, *SYK*, *FYN*, proteomics, gene expression, RNAseq

## Abstract

**Background:**

Oncogenic mechanisms in small-cell lung cancer remain poorly understood leaving this tumor with the worst prognosis among all lung cancers. Unlike other cancer types, sequencing genomic approaches have been of limited success in small-cell lung cancer, i.e., no mutated oncogenes with potential driver characteristics have emerged, as it is the case for activating mutations of epidermal growth factor receptor in non-small-cell lung cancer. Differential gene expression analysis has also produced SCLC signatures with limited application, since they are generally not robust across datasets. Nonetheless, additional genomic approaches are warranted, due to the increasing availability of suitable small-cell lung cancer datasets. Gene co-expression network approaches are a recent and promising avenue, since they have been successful in identifying gene modules that drive phenotypic traits in several biological systems, including other cancer types.

**Results:**

We derived an SCLC-specific classifier from weighted gene co-expression network analysis (WGCNA) of a lung cancer dataset. The classifier, termed SCLC-specific hub network (SSHN), robustly separates SCLC from other lung cancer types across multiple datasets and multiple platforms, including RNA-seq and shotgun proteomics. The classifier was also conserved in SCLC cell lines. SSHN is enriched for co-expressed signaling network hubs strongly associated with the SCLC phenotype. Twenty of these hubs are actionable kinases with oncogenic potential, among which spleen tyrosine kinase (SYK) exhibits one of the highest overall statistical associations to SCLC. In patient tissue microarrays and cell lines, SCLC can be separated into SYK-positive and -negative. *SYK *siRNA decreases proliferation rate and increases cell death of *SYK*-positive SCLC cell lines, suggesting a role for *SYK *as an oncogenic driver in a subset of SCLC.

**Conclusions:**

SCLC treatment has thus far been limited to chemotherapy and radiation. Our WGCNA analysis identifies SYK both as a candidate biomarker to stratify SCLC patients and as a potential therapeutic target. In summary, WGCNA represents an alternative strategy to large scale sequencing for the identification of potential oncogenic drivers, based on a systems view of signaling networks. This strategy is especially useful in cancer types where no actionable mutations have emerged.

## Background

Small-cell lung cancer (SCLC) represent up to 15 % of lung cancers and pose a major challenge as we are unable to diagnose it early, its most aggressive clinical behavior and the lack of lasting benefit from therapy. Patients presenting with this neuroendocrine tumor of the lung have a dismal 5% 5-year survival rate. Although SCLC is highly sensitive to chemotherapy and radiation, it invariably recurs with fatal widespread metastasis [1]. In contrast to non-small cell lung cancer (NSCLC), to date no specific genetic biomarkers or molecular subtypes have been identified in SCLC [2]. Gene expression profiling has had limited success in SCLC stratification for the purpose of personalized treatment. Although recent advances in genomic analysis of SCLC have identified potential driver mutations in SCLC [3-5], there remains an unmet need for approaches that can stratify SCLC patients and/or uncover viable molecular targets in SCLC.

To meet this challenge, we turned to weighted gene co-expression gene network analysis (WGCNA), a recently introduced bioinformatics method that captures complex relationships between genes and phenotypes. The distinct advantage over other methods, such as differential gene expression, is that WGCNA transforms gene expression data into functional modules of co-expressed genes without any prior assumptions about genes/phenotypes, providing insights into signaling networks that may be responsible for phenotypic traits of interest [6-8]. In lung cancer, its potential remains unexplored.

Our WGCNA analysis of a public lung tumor dataset [9] revealed a module of co-expressed genes specific to SCLC. After filtering, the SCLC-specific module was reduced to a SCLC-specific hub network (SSHN) signature that classified SCLC from other lung cancer types in several public and in-house tumor datasets (including independent high-throughput screening techniques such as RNAseq and shotgun proteomics), and in lung cancer cell lines. SSHN was enriched for hubs in signaling networks known to be associated with SCLC pathogenesis, including cell cycle, oxidative stress response and DNA damage response. As a proof of concept, we chose to validate oncogenic kinase hubs (20 kinase genes) within SSHN, as they provide special translational relevance as potential candidates for targeted therapy and also play key roles in various hallmarks of cancer. Among the twenty, spleen tyrosine kinase (*SYK*), a previously undescribed target in SCLC, exhibited one of the highest overall statistical associations with the SCLC phenotype, based on WGCNA gene significance (GS, see Methods) and overexpression in shotgun proteomics, and was therefore selected for further validation as a target.

*SYK *has been previously investigated most extensively in the context of lymphocyte development and as a therapeutic target in hematologic malignancies. SYK activation leads to several downstream events that promote cell survival, including activation of phosphatidylinositol 3-kinase (PI3K) and AKT, and the phosphorylation of multiple signaling proteins [10-12]. In B-cells, it transduces tonic signaling by physical interaction with the immunoreceptor tyrosine-based activation motif (ITAM) of the B-cell antigen receptor (BCR) complex [13], positively regulating survival and proliferation during development and immune response. SYK is also associated with the Fc receptor in B-cells, which instead has opposite effects to the BCR [14,15]. The balance of regulation on survival and proliferation downstream of SYK is influenced by redox signaling: NADPH oxidase, in close proximity to BCR, can produce peroxide that inhibits phosphatase action on BCR-activated SYK, reinforcing tonic signaling [16]. Another important function of SYK is response to oxidative stress where SYK gets activated and promotes pro-survival pathways [17]. B-cells die in response to *SYK *knock-down and fail to develop in SYK-deficient mice [15]. Together, these observations have formed a rationale for SYK-targeted therapy in hematological malignancies with small molecule kinase inhibitors [12,18,19]. SYK has not been studied in the context of lung neuroendocrine (NE) cells, the SCLC cells of origin, whose oxygen sensing functions, in analogy with BCR, rely on redox signaling [20].

To our knowledge, *SYK *has not been proposed before as an oncogenic driver or candidate target in SCLC. Based on our WGCNA results, we investigated this possibility. We determined that 11 out of 33 SCLCs were SYK-positive by immunostaining in patient tissue microarrays (TMAs). Moreover, SYK knock-down reduced proliferation and survival in SYK-positive SCLC lines. We propose that *SYK *is an oncogenic driver in SCLC and that SYK expression may be developed as a companion biomarker for SYK targeted therapy.

## Results

### Identification of a SCLC-specific co-expression module

**Figure 1:**
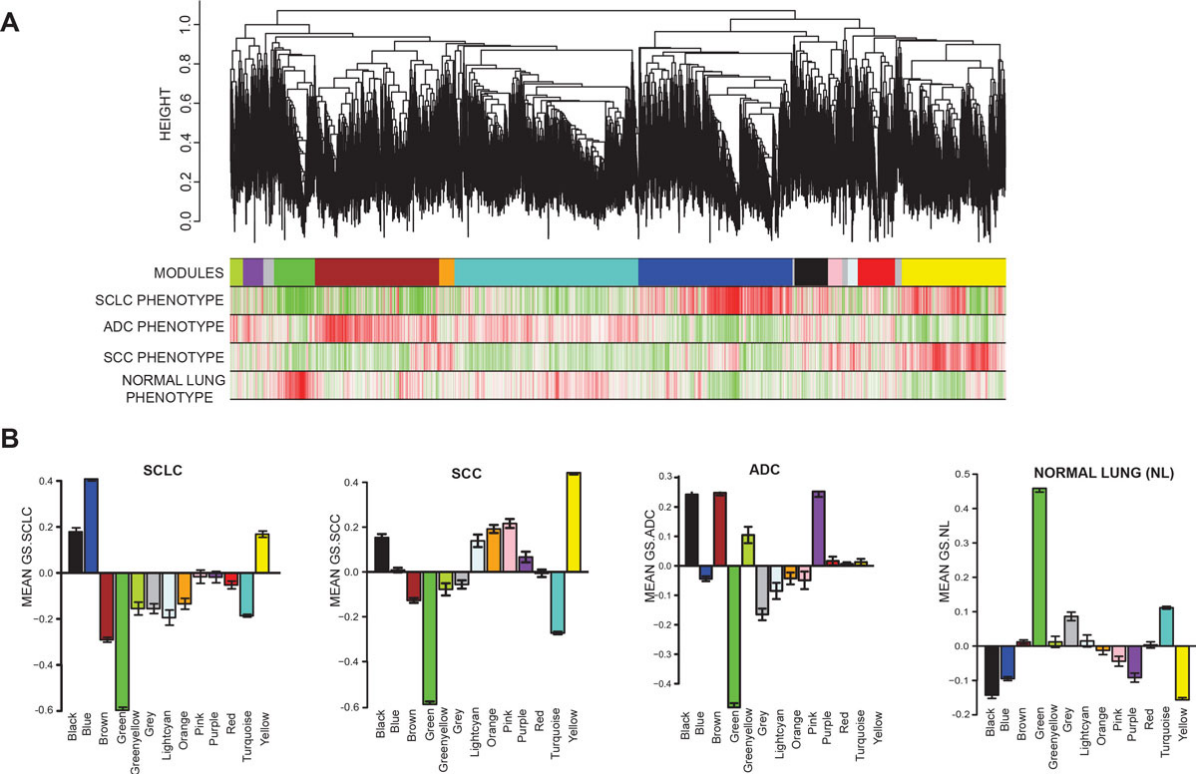
**Identification of SCLC-specific modules using WGCNA**. (A) In the hierarchical dendrogram, lower branches correspond to higher co-expression (height = Euclidean distance). The 13 identified modules were coded by the colors indicated below the dendrogram. Below, red and green lines indicate positive or negative correlations, respectively, with lung tumor types on the left. (B) Average 'gene significance' (GS) of genes within a specific module summarized in the barplot for each lung tissue type (left to right: SCLC, SCC, ADC, and NL). The blue module is associated solely with SCLC.

To identify a hierarchical network view of co-expressed genes across lung cancer subtypes, we applied WGCNA to a public dataset (GEO ID: GSE6044 - 33 untreated patients) comprised of 5 normal, 9 adenocarcinoma (ADC), 9 squamous cell carcinoma (SCC) and 9 SCLC lung cancer tissue specimens [9]. An unsupervised correlation similarity matrix was built based on pairwise correlations between genes. Unsupervised average linkage hierarchical clustering of all genes in this dataset resulted into 13 modules (Figure 1A) labeled by color and each comprised of mutually exclusive co-expressed genes. Genes with no distinct module assignment are grouped in a grey module by WGCNA. None of these modules were identified using any pre-assigned phenotype or gene bias. To ensure that modules were not being detected by chance, we simulated a random dataset containing same number of samples and genes as our test dataset. Only two modules were generated from the random dataset, turquoise and grey (with the grey module containing the vast majority of genes), indicating that WGCNA module identification in our test dataset is in fact driven by meaningful gene co-expression patterns (Additional file 1, Figure S1).

Following the unsupervised module generation, individual gene correlations to a specific phenotype (normal lung, ADC, SCLC, SCC) were quantified by gene significance (GS). The average GS of all genes within each module is summarized in Figure 1B. This analysis unveiled positive or negative correlation of certain modules with specific lung cancer subtypes, or normal lung. The brown and purple modules appeared to be ADC specific, and included previously identified ADC markers cytochrome B5 (*CYB5A*) or surfactant protein B, C and D (*SFTPB, SFTPC, SFTPD*), respectively[21]. Yellow, pink, orange and light cyan modules were SCC specific and included involvulin (*IVL*), cytokeratin 14 (*KRT14*), and galectin-7 (*LGALS7*) [21-23] (Additional file 2). The green module contained genes positively correlated to the normal lung phenotype and negatively correlated with all tumor subtypes (SCLC, ADC, and SCC), making it a "normal lung module" (Additional file 2).

The blue module was specific to SCLC (Figure 1). Accordingly, it contained genes that have already been associated with SCLC progression such as Achaete-scute complex homolog 1 (*ASCL1*), Neural cell adhesion molecule 1 (*NCAM1*/*CD56*), Thyroid transcription factor-1 (*TTF-1*) and Insulinoma associated-1 (*INSM1*) [24,25] (Additional file 2).

### Identification and validation of a SCLC-specific hub network (SSHN) of co-expressed genes across genomic and proteomic platforms

To identify and validate a network of co-expressed genes that is specific to SCLC, we focused on the blue module. The SCLC-specific blue module (1696 genes; Figure 1) is comprised of co-expressed up-regulated genes across SCLC tumors. Each module is arranged in the form of a hierarchical network (due to hierarchical clustering used to obtain the modules, Figure 1A dendrogram). Therefore, each module consists of a few highly connected "hubs" (genes that have high intramodular connectivity kME) as well as many genes with fewer connections. The rationale behind building hub-based networks is to narrow down the list of relevant candidates, based on the assumption that highly connected hubs are more vulnerable targets to alter network performance. This assumption has been successful in several examples from biological networks in yeast [26,27] and mammalian cells, including cancer [6,28].

Each module can be further filtered to identify the top hubs relative to desired criteria using measures such as intramodular connectivity (kME) and gene significance (GS) [29]. We filtered the blue module genes to obtain hubs that ranked high in each of the following criteria: a) high positive correlation with SCLC phenotype given by gene significance (GS.SCLC >0.5); b) high intramodular connectivity (blue module kME >0.5); and c) high T-test statistic (overexpression in SCLC versus normal lung > 5) and a p-value less than 0.01. This filtering approach produced 287 hub genes, which are not only overexpressed in SCLC, but also highly connected within SCLC. We refer to this network of 287 hubs as *SCLC-specific hub network *(SSHN) (Additional file 3).

**Figure 2:**
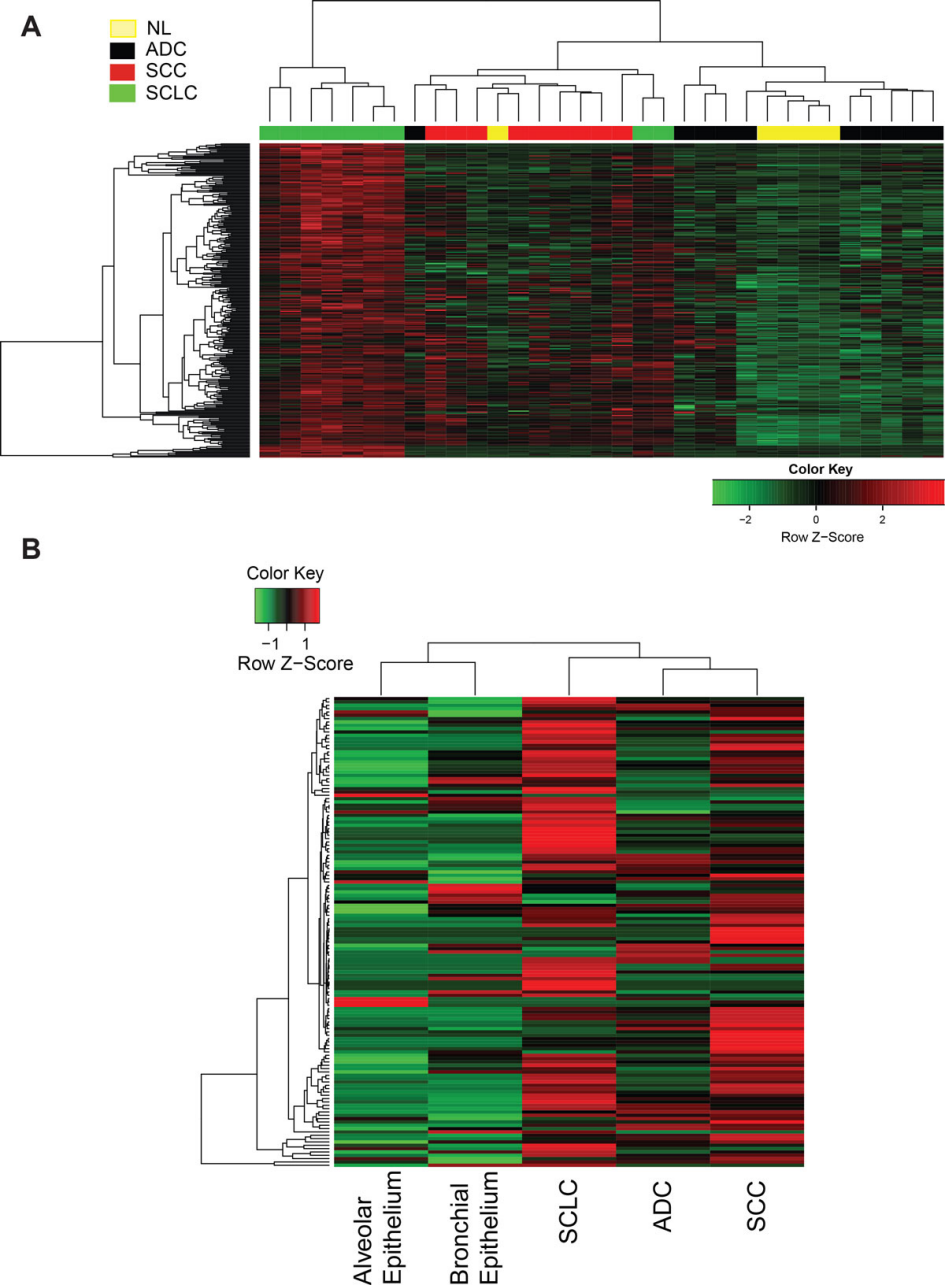
**Validation of SSHN as a robust classifier for SCLC in two independent datasets from (A) high-throughput gene expression and (B) shotgun proteomic analysis**. (A) Unsupervised clustering heatmap based on 287 SSHN genes (rows) of lung cancer patients (columns) in GSE6044 dataset [9]. Red and green indicate high and low expression, respectively. The majority of SCLCs cluster by themselves on the far left of the dendrogram. Two SCLC specimens are excluded from this cluster, a trend to be investigated in more depth if confirmed in larger datasets (see Discussion). (B) SSHN-based unsupervised clustering heatmap of an in-house generated shotgun proteomic dataset comprised of control alveolar and bronchial epithelium, ADC, SCC and SCLC tissue specimens (for each tissue type, specimens from multiple patients, five in this case, were pooled as it is customary for shotgun proteomic analysis). Red and green as denoted in (A). Analysis is limited to 141 out of 287 SSHN proteins (rows), since the remainder proteins were not detect by shotgun proteomics. The 3 tumor specimens segregate together from normal tissue. Within the 3 tumor specimens, ADC and SCC are more similar to each other than to SCLC.

To validate the robustness of SSHN as a SCLC-specific classifier, it was first applied by unsupervised hierarchical clustering bootstrap analysis to patient samples in a test public dataset (GSE6044) from which the blue module was derived. The SSHN classified SCLC away from every other lung tumor subtype (ADC and SCC) and normal lung, the area under ROC curve (AUC) was 0.87 with 95% confidence interval (CI) of [0.72, 1] (Figure 2A). The performance of the SSHN classifier was reproducible in both an independent validation patient dataset of 163 tumors (GSE11969) [30] generated in a different array platform (Agilent) (AUC of 1) (Additional file 1, Figure S2A), as well as in our own microarray dataset containing 23 SCC and 10 SCLC samples (AUC of 0.94 with 95% CI of [0.85, 1])(Additional file 1, Figure S2B). In the GSE11969 dataset, the SSHN also proved to be an excellent classifier for distinguishing SCLC from large cell carcinoma (LCC) subtype (Additional file 1, Figure S2A). Interestingly, large cell neuroendocrine carcinomas (LCNC), another high-grade neuroendocrine tumor (NET) of the lung, co-clustered with SCLC, confirming similarities between the 2 tumor types as reported previously [31]. On all the three patient datasets, the SSHN genes are highly predictive of SCLC against other tissue types with statistically significant p-values less than 0.0001.

To further validate the SSHN as a classifier, we used next-generation sequencing to produce genome-wide RNA-seq data on an independent set of tissues including 10 SCLCs, 5 SCCs, and 5 normal lung tissue specimens. We detected overexpression of 206 genes out of 287 SSHN genes that differentiate SCLC (71.8%) from normal lung alone (at 5% FDR) while 106 genes out of 287 SSHN genes differentiate SCLC (71.8%) from normal lung and SQCC (at 5% FDR) (Additional file 3), indicating that SSHN is a robust classifier in another data type (RNA-seq).

Finally, the SSHN gene expression classifier was further validated at the protein level in yet another in-house, independent set of formalin fixed paraffin embedded patient tissue samples analyzed by shotgun proteomics and comprised of 5 samples each of SCLC, SCC, ADC and age- and smoking history-matched normal lung tissues specimens, pooled by histologic type. Out of 287 SSHN genes, 141 gene products were detected at the proteomic level and also classified the SCLCs apart from the other tissues (Figure 2B). To our knowledge, this is a first report of an entire SCLC genomic signature validated at the proteomic level.

In each of the 4 datasets, there were 1-2 specimens that did not segregate with the SSHN-defined SCLC cluster, but were clinically diagnosed as SCLC (Figure 2A; Additional file 1, Figure S2). This could be due to mis-diagnosis as is fairly common in SCLC due to mixed SCLC-NSCLC histology [32], or possibly a small subset of patients whose tumors have different biology. Overall, we conclude that the SSHN is a robust molecular classifier to distinguish SCLC from other lung tumor types and normal lung across multiple gene and protein expression platforms.

### Biological insights from the SSHN: Network enrichment analysis and target identification

To gain biological insights in SCLC biology, the SSHN component genes were further categorized into functional pathways based on the assumption that they are co-upregulated because of shared cellular functions. Analysis of SSHN by Webgestalt [33] revealed that SSHN is enriched for functional pathways summarized in Additional files 4 and 5 and Figure S3 in Additional file 1, and include cell cycle and checkpoint response (total of 25 genes), cellular stress response (41 genes of which 21 genes related to oxidative stress), and DNA damage response and repair pathways. All p-values were adjusted for multiple comparisons in Webgestalt and therefore effectively rank the significance of these functional pathways in SCLC phenotype.

**Figure 3:**
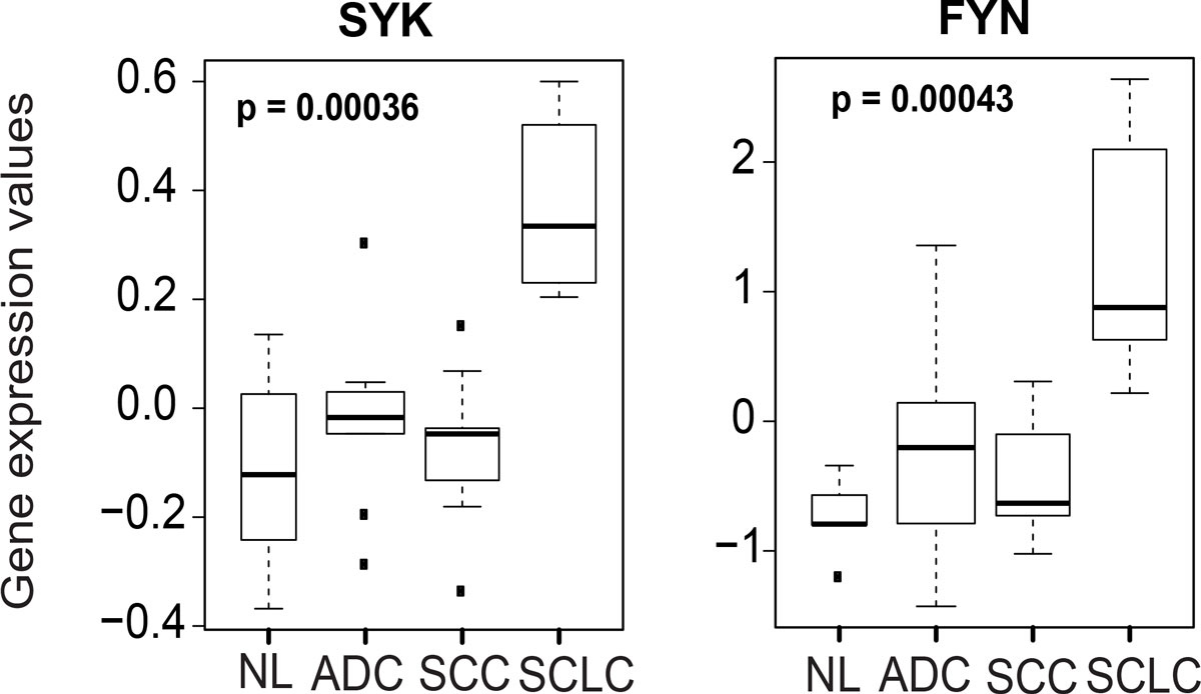
**Co-expression of 2 SSHN kinases FYN and SYK in SCLC patients**. Log2 expression values are indicated in the boxplots for each individual hub within SSHN across various patient lung tissues from the GSE6044 test dataset [9]. The outliers are denoted by dots. P-value shows statistical significance by Kruskal-Wallis nonparametric test [81]. FYN and SYK are co-overexpressed in SCLC patients versus NSCLC (ADC, SCC) and normal lung.

As a proof-of-concept that connected hubs identified by WGCNA are of biological relevance, we further refined the pathway analysis by focusing on kinases, since these tend to be of the greatest translational value. There were 20 kinases contained in the SSHN (Additional file 6), all worth investigating in the context of SCLC. However, shotgun proteomics data (available for 4 kinases, Additional file 6) indicated that *SYK *is strongly overexpressed within the SCLC phenotype compared to normal tissue (high "SCLC vs. Bronchial epithelium Rate ratio" and "SCLC vs. Alveolar epithelium Rate ratio", column J and M in Additional file 6, respectively). *SYK *is an oncogenic non-receptor tyrosine kinase involved in hematologic malignancies [12,18,19]. Another oncogene, the SRC-family kinase *FYN*, was also part of this SSHN kinase set. SYK is an intracellular signal transducer downstream of growth factor/T-cell/B-cell receptors well known to work in concert with SRC-family kinases [15]. Specific overexpression of SYK and FYN in SCLC, compared to other lung tumor types, has not been previously reported, to the best of our knowledge (Figure 3). Together, these clues prompted us to select *SYK *and *FYN *for further investigation in the context of SCLC tumors.

**Figure 4:**
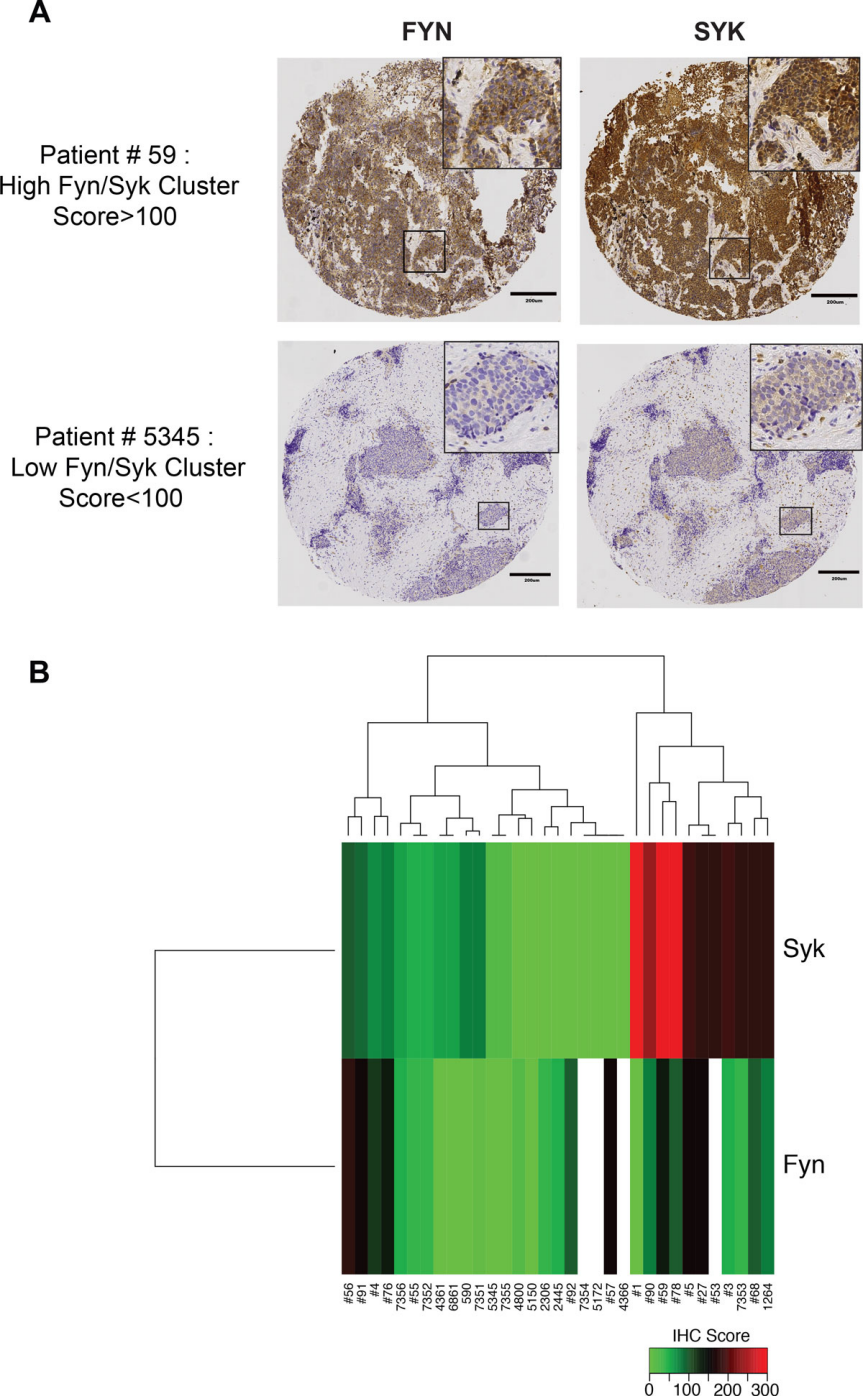
**Co-expression of SYK and FYN in a subset of SCLC tumors**. Contiguous sections of TMAs from 39 SCLC patient specimens were stained with antibodies to SYK and FYN, respectively. Stained sections were scored by a pathologist as described in Methods. (A) Representative stained sections showing positive (upper) or negative (lower) results. See text for additional details. Tumor spot images were captured by brightfield microscopy at 20X magnification. (B) Unsupervised hierarchical clustering heatmap of SYK and FYN immunostaining intensity scores across SCLC patients distinguished positive from negative tumors as described in Methods. Red and green indicate high and low expression, respectively. Specimens that are positive for both SYK and FYN segregate in one cluster, on the right. Patient ID shown below the heatmap.

To verify co-expression at the protein level, we immunostained for SYK and FYN in a panel of SCLCs assembled in tissue microarrays (TMAs). All specimens were tested in duplicate, and the expression of SYK and FYN consistently co-varied (Figure 4A), with a correlation of 0.28 across SCLC specimens. Clustering analysis of the staining scores of SYK/FYN expression separated the TMA specimens into 2 groups, SYK/FYN-positive and -negative tumors (Figure 4B).

### Preservation of SSHN and differential SYK/FYN expression in SCLC cell lines

**Figure 5:**
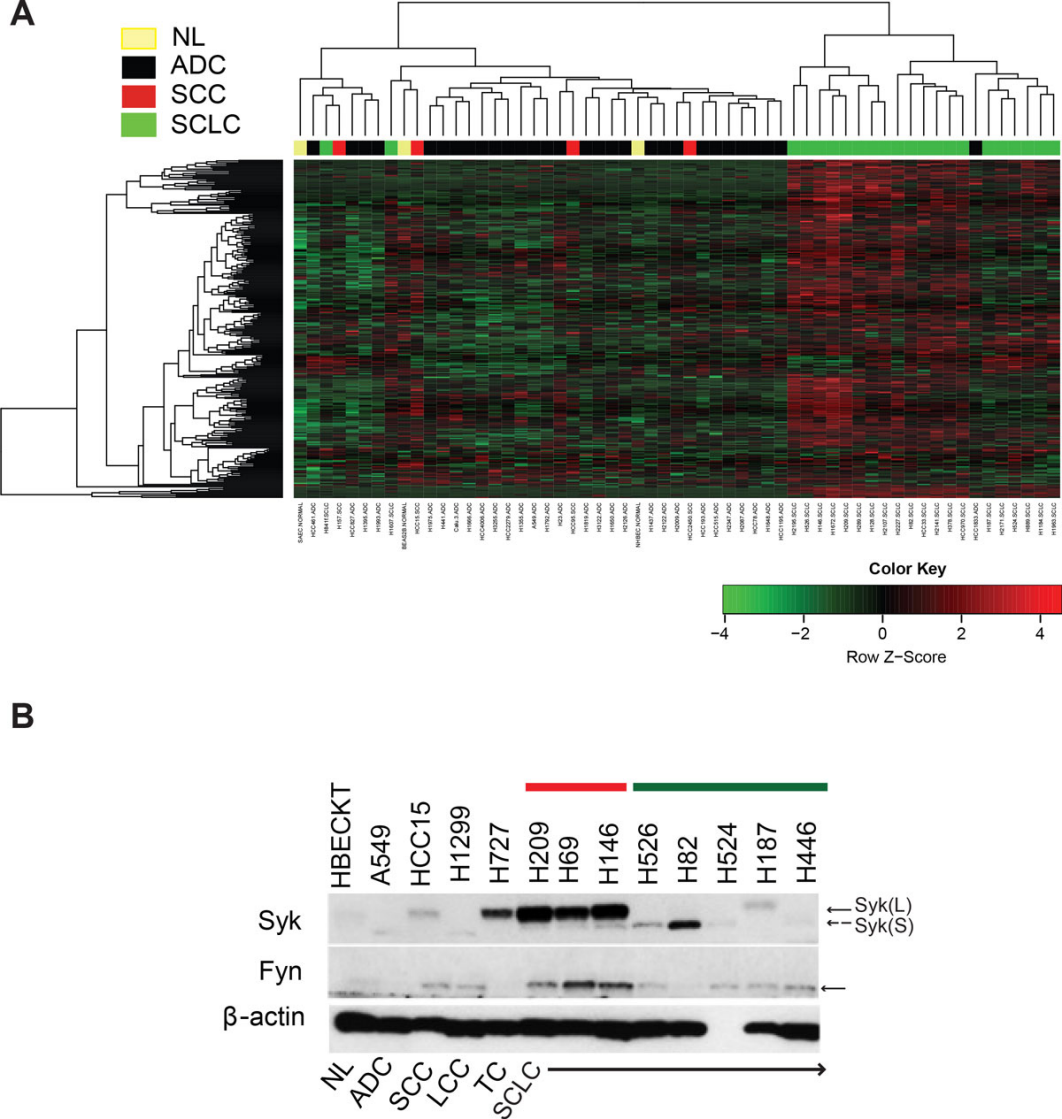
**SSHN is preserved in SCLC cell lines**. (A) Unsupervised clustering heatmap based on SSHN genes (rows) of lung cancer cell lines (columns) in GSE4824 dataset [36]. Red and green colors in rows of the heatmap indicate high and low expression respectively. This analysis shows SSHN conservation across SCLC cell lines. (B) Representative western blot of SYK and FYN in various lung cancer cell lines. FYN and SYK are selectively overexpressed in SCLC cell lines. Within the SCLC cell lines, the red and green bars indicate FYN/SYK-positive and -negative SCLC cell lines, respectively. Arrows point to bands corresponding to the expected molecular weight for SYK and FYN. The dotted arrow indicate the position of a shorter form of SYK protein (SYKB or S) that lacks 23 amino acids [37].

SYK and FYN are attractive candidates for targeted therapy [34,35]. To test their functional relevance in SCLC, we turned to SCLC cultured cell lines. The SSHN classifier was conserved in a large panel of lung cell lines [36]. As indicated by clustering analysis (Figure 5A), 21 out of 23 SCLC cell lines separated nicely from the other 36 lung cancer cell lines tested (AUC of 0.97 with 95% CI of [0.94, 1]). Note that 2 SCLC cell lines did not follow this pattern, an observation mirrored in tumor specimens (Figure 2; Additional file 1, Figure S2) that warrants further studies.

We investigated co-expression of SYK and FYN in SCLC cell lines by western blotting of whole-cell lysates with appropriate antibodies (Figure 5B). Similar to our protein expression shown by immunostaining of our TMAs, SYK and FYN exhibited a trend to co-vary in SCLC cell lines (Figure 5B), opening an avenue to biochemical analyses of the functional value of this differential expression. Note that SYK has two splice-variant isoforms - long (L or p72^SYK^) and short (S or B) that lacks 23 amino acids [37]. The SYK positive cell lines overexpress SYK (L) form while other cell lines express low or no SYK (S) (Figure 5B).

### Inhibiting SCLC cell line viability by SYK knock-down

**Figure 6:**
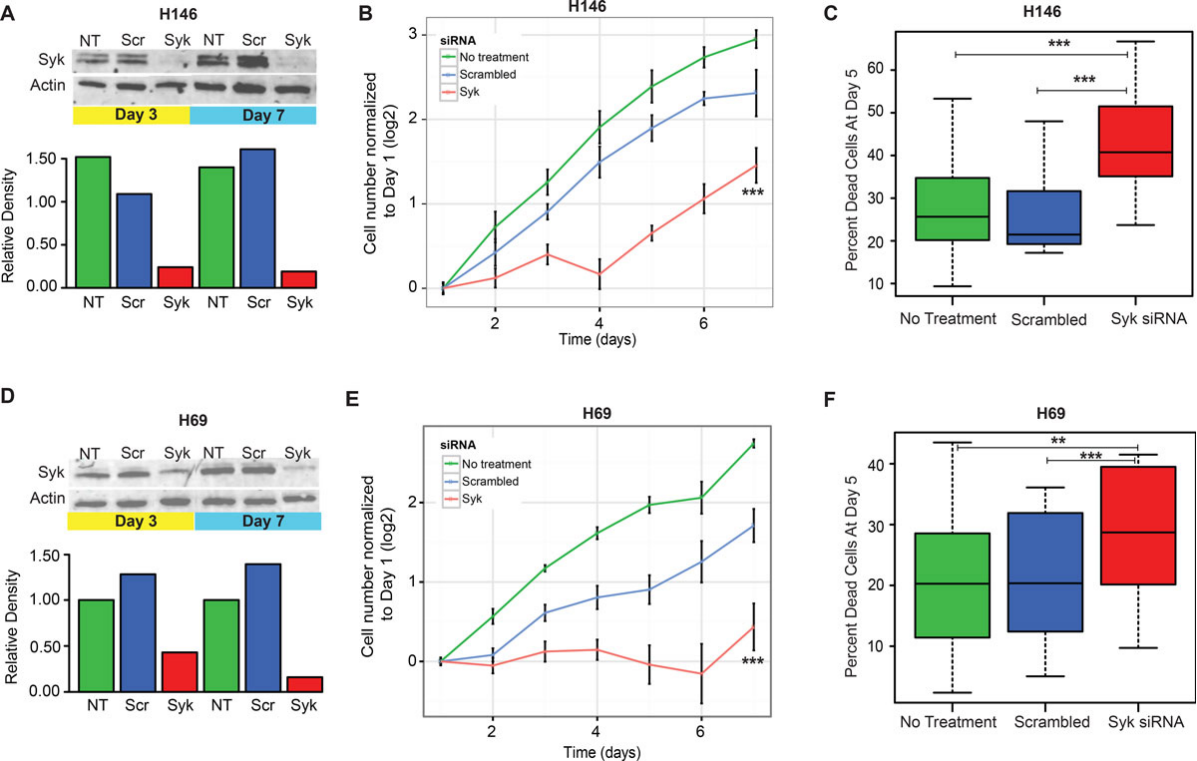
**Effect of Syk knock-down in Syk/Fyn positive SCLC cell lines**. The SCLC cell lines H146 (A-C) and H69 (D-F) were treated with Syk-specific and control siRNA as described in Materials and Methods section. (A, D) The efficiency of inhibition was measured by Western blotting on day 3 and 7 post transfection. Syk resolves as two bands, of which the lower is a less-functional splice variant that lacks 23 amino acids [37]. Band intensity (lower panels) was quantified by densitometry in ImageJ (http://rsbweb.nih.gov/ij/). (B, E) Cell proliferation, measured by cell counts as described in Materials and Methods section, shows that Syk-siRNA treatment induces statistically significant growth inhibition compared to untreated cells and to scrambled siRNA treatment. Asterisks denote overall statistical significance of slope as compared to control across the siRNA conditions, as follows: <0.0005 '***' 0.001 '**' 0.01 '*' 0.05 '.'. The viability growth curves (from N = 4 experiments) statistics were generated from slopes of a linear regression model. Multiple comparison of treatments were derived using ANOVA and Tukey's method [80]. (C and F) Percentage of dead cells (percent of ethidium homodimer positive cells normalized to total cell counts, see Materials and Methods) is significantly higher (H69 p-value < 2.2e-16; H146 p-value < 2.2e-16) in Syk siRNA treated cells at day 5, compared to controls. Asterisks denote statistical significance measured by paired *t*-test as compared to control across the siRNA conditions, as follows: <0.0005 '***' 0.001 '**' 0.01 '*' 0.05 '.'.

To assess the validity of SYK and/or FYN as targets in SCLC, we down-regulated the expression of these proteins using siRNA in the H69 and H146 cell lines (Figure 5B). siRNA induced 80-90 percent reduction in total protein expression for each of these molecules in both H69 and H146 (Figure 6A and 6D; Additional file 1, Figure S5A and D). We assessed viability with automated microscopy, imaging-based methods (Live-dead assay, see Methods; images and segmentation for obtaining cell counts shown in Additional file 1, Figure S4). SYK knock-down caused a significant decrease in proliferation rates compared to scrambled control in both H69 and H146 (Figure 6B and 6E), while FYN knock-down showed little effect (Additional file 1, Figure S5B and E). The decrease in proliferation was in part due to a loss of cell viability, as indicated by increased cell death by Day 5 in SYK knock-down cells assessed by ethidium homodimer positivity (Figure 6C and 6F; Additional file 1, Figure S5C and F). Together, these data suggest that SYK is a candidate therapeutic in SYK/FYN-expressing SCLCs.

## Discussion

We report several findings of immediate translational value for SCLC: 1) derivation of an SCLC-specific hub network (SSHN) that classifies SCLC from other lung cancers, including the closely related neuroendocrine tumors; 2) validation of the SSHN classifier across many data types, including expression microarrays from multiple platforms, RNAseq and shotgun proteomics; 3) co-varied expression of 2 oncogenes, *SYK *and *FYN*, in a subset of SCLC tumors and cell lines; and 4) identification of SYK as a candidate biomarker and therapeutic target for SCLC.

The increasing availability of large gene expression cancer datasets presents unprecedented opportunities for translational advances. Challenges in data analytics, however, must be met. For instance, the predominant metric of differential gene expression is silent on disease relevance of identified gene products, since it provides no measure of their functional relatedness [38], and its resulting signatures do not replicate well across datasets [7,39]. The number of potential therapeutic targets (e.g., ranked by differential expression scores) is large and expanding, but target prioritization is hampered by lack of functional insight. In contrast, analyses based on gene co-expression algorithms perform well across data types [7] and inspire working hypotheses since their results resemble hierarchical signaling networks. Accordingly, the SCLC-specific co-expressed gene classifier network SSHN we report here is robust across datasets encompassing different types of lung cancer (Figure 2; Additional file 1, Figure S2; Additional file 2). In particular, despite being derived from gene expression microarray data, the SSHN performed well on proteomic lung cancer specimens. Note that each of the datasets tested were obtained from independent SCLC patient cohorts. To our knowledge, this is the first report of signature preservation on a shotgun proteomic SCLC dataset. Other co-expression based approaches have also been successfully applied in other cancers such as breast cancer [7].

Neuroendocrine lung tumors, to which SCLC belong, are sometimes difficult to sort out based solely on the current World Health Organization (WHO) criteria of morphology and mitotic rate, warranting searches for additional biomarkers [32,40,41]. The SSHN signature begins to address this need, e.g., distinguishing SCLC that stain negative for neuroendocrine markers such as synaptophysin and chromogranin A (~25%) [25,32] from NSCLC, and mixed SCLC-NSCLC from NSCLC. However, because of the very small number of LCNEC samples studied by gene expression analysis, we cannot exclude the possibility that other LCNEC tumors would co-cluster with SCLC. In addition, due to the lack of larger SCLC datasets and the limited clinical information on the available SCLC datasets, careful validation of our results, including outcome associations, is definitely warranted.

While SSHN as a whole is an effective SCLC classifier, its individual component genes (or gene products) may or may not be expressed in a particular tumor. This is not at all surprising, due to the expected inter-tumor heterogeneity within a particular histological type [32]. Our data suggest that within the SCLC cluster defined by SSHN, a further subdivision between SYK/FYN-positive and -negative may be informative. A few specimens classified as SCLC by pathological and clinical criteria, did not cluster with SSHN-defined SCLC (Figure 2A; Additional file 1, Figure S2). Whether these are misdiagnosed or represent disease heterogeneity or different stage of tumor progression remains to be tested.

Receptor and non-receptor tyrosine and serine-threonine kinases are effective actionable targets in cancer. SSHN contains twenty kinases and growth factor receptors, including *TTK, TLK2, NEK2, CDK4, FYN, PLCG1, SYK *(Additional file 6). None of these were previously reported in SCLC; thus, prioritization strategies are called for. The kinases *SYK *and *FYN *stand out as potential SCLC targets for several reasons. Besides being tightly associated with the SCLC phenotype, they are already proven as candidate targets in other cancers, such as CML [10,42], AML [12], retinoblastoma [43], glioblastoma [44] and prostate cancer [45,46]. They also activate Focal adhesion kinase (FAK) [47,48], previously shown by our group to be amplified, overexpressed and constitutively activated in SCLC [49,50]. They play key roles in anchorage independence, survival and oxidative stress response by activating multiple downstream pathways including AKT and ERK kinases [15,35].

We found that SYK knock-down significantly decreased viability and growth rates in SYK/FYN-positive SCLC via increased cell death (Figure 6), suggesting that *SYK *plays an oncogenic driver role and that inhibitors could potentially be used in SYK-positive SCLC, alone or in combination with chemotherapy. Increased cell death was also observed in AML via knock-down of SYK [12]. Further studies are needed to discriminate between overexpression versus activation of SYK in SCLC.

Our findings unveil an unsuspected link between SCLC and the biology of B-cell leukemias/lymphomas that is worth exploring. The role of SYK in B-cell receptor (BCR) initiated tonic signaling both in normal B-cells and lymphomas is well established [14,15]. Tonic signaling promotes proliferation and survival of B-cells. Mice lacking SYK exhibit profound B-cell development deficits, and die embryonically from severe hemorrhages, also pointing to indispensable SYK signaling in cell types other than B-cells [51]. Targeted SYK therapy has been advocated in various types of B-cell lymphomas, and specific inhibitors for its kinase activity are already approved such as R406, fostamatinib [14,18,19,34], opening avenues for testing targeted treatment in SCLC. SYK signaling in NE (and possibly SCLC) may be associated with oxygen sensing [20], but SYK-associated receptor(s) in NE or SCLC cells remain to be defined.

There are several reports of tumor suppressor functions for *SYK *in several solid tumor types, including breast cancer [52], gastric cancer, and melanoma [53]. Additional data are needed to reconcile these seemingly conflicting roles of *SYK *as oncogene or tumor suppressor. In this regard, it is worth noting that in B-cells effects of SYK on survival and proliferation are modulated by associated SRC-family kinase members [13]. Differential interactions of SYK with such kinases in a tumor-specific manner are a possible explanation for the dual role of *SYK *as a tumor suppressor in some cancers [52,53], and an oncogene in hematologic malignancies [10,12] and SCLC. Therefore, an immediate priority is to determine the type of receptor SYK is associated with in SCLC, and its possible regulation by SRC-family kinases such as FYN (see below).

In agreement with our results, in the Cancer Cell Line Encyclopedia [54], 35 out of 49 SCLC cell lines tested overexpress SYK (> 2 fold of the median centered intensity values). In another recent large dataset 33 of 53 SCLC cell lines overexpress SYK [55]. We confined our experimentation to SCLC cultured cell lines and knock-down of SYK expression. While our data are encouraging, future studies should address applicability to spontaneous [56] or human xenotransplant mouse models of SCLC [57]. Furthermore, it remains to be seen whether inhibition of SYK-kinase activity, in addition to expression, elicits a death response in SCLC.

It is worth noting that to date no SYK mutations have been reported in any tumor type. SYK gene fusions or translocations have been reported in hematologic malignancies, in which a driver function for overexpressed SYK has also been postulated [15,58,59]. On the other hand, SYK negative tumors have hypermethylation and loss of function of the SYK gene [60]. Thus, the biology of SYK-positive SCLC tumors may be potentially distinct from SYK-negative SCLC tumors, with differences due to stages of progression, or divergence of transforming mechanisms.

SYK signaling functions are mediated in concert with SRC-family kinases [15]. This subject is not fully understood and, in particular it is not clear to what extent various SRC-family kinases are interchangeable in this role within a given cell type. It is perhaps not coincidental that a SRC-family kinase, FYN, was identified in the blue module by WGCNA and that a strong co-expression correlation was found in SCLC TMAs and cell lines (Figure 4 and 5). Byers.et.al also reported activation of SRC-family kinases in SCLC assessed via reverse phase protein arrays (RPPA) [61]. On the other hand, FYN kinase inhibition had no effect on SCLC cell line survival (Additional file 1, Figure S6). Clarifying the SYK-FYN signaling connection in SCLC, and the possible redundancy of SRC-family kinases may open avenues to productively deploy inhibitory combination of SYK and FYN targeted therapy.

In the TMA patient dataset, we detected 2 groups of SCLC based on SYK/FYN expression alone (Figure 4A and 4B). Admittedly, this dataset is too small to reach conclusions, highlighting the need for larger patient populations. Nonetheless, our observations raise the possibility of distinct treatment strategies in SYK-positive SCLC tumors, by analogy to lung tumors overexpressing EGFR, or HER2+ breast cancers, whose response to targeted therapy dramatically improves the outcome [62,63].

Here we have implemented an alternative strategy to large scale sequencing, based on a systems view of signaling networks provided by gene co-expression analysis. We respectfully submit that this approach can provide useful translational insights in the biology of specific cancer types.

## Conclusions

We have identified a robust co-expression network based signature (SSHN) for SCLC tumors on three independent platforms (microarrays, RNAseq and shotgun proteomics). This signature was also conserved in SCLC cell lines. Within this SSHN network, we found twenty targetable kinases that were overexpressed in most, if not all of these platforms. Two tyrosine kinases SYK and FYN were overexpressed significantly in SCLC patients and cell lines by several independent bioinformatics and experimental methods, and distinguished between two potential groups of patients - SYK/FYN positive and negative. The SYK/FYN positive SCLC cell lines exhibited significant loss of viability and increased cell death in response to SYK siRNA, providing evidence for SYK as a novel oncogenic driver for SCLC. All SCLC patients get treated with combination chemotherapy (cisplatin, etoposide) without distinction. Our work suggests that stratifying patients with respect to SYK/FYN expression may open avenues to personalized medicine in SCLC, given that SYK small-molecule inhibitors are already in clinical trials for other disease conditions. Future work will have to determine whether in fact SYK may represent a potential actionable target in SCLC, by itself or in combination with chemo or radiation therapy.

## Methods

### Cell lines and reagents

All normal, NSCLC and SCLC cell lines were purchased from ATCC (http://www.atcc.org). All lung cancer cell lines were grown in RPMI containing 10% fetal bovine serum (GIBCO^®^) as recommended by ATCC with the exception of HBECKT (Keratinocyte serum free media). SYK, FYN and beta-Actin (Sigma-Aldrich^®^) antibodies were used for western blotting and IHC.

### Microarray data normalization

Public datasets on the Affymetrix platform (GSE6044) [9], GSE4824 [36]) were downloaded from GEO [64] as CEL files, normalized and median centered using quantile RMA normalization using Affy Bioconductor package [65] in R [66]. Agilent datasets, GSE11969 [30] and our own Agilent dataset, were Lowess-normalized and median centered using GeneSpring [67].

### Network analysis

Probe-level data for all the datasets was converted to gene-level data by probe merging using the collapseRows function [68]. Probes with no known gene symbols were removed from further analyses to reduce the dimensionality of the dataset. The co-expression network analysis was performed in R using the WGCNA package as previously described [6,8]. Briefly, all genes in the training dataset (GSE6044) were used to build unsupervised co-expression based similarity matrix using Pearson's correlation coefficient. The similarity matrix was converted to a weighted adjacency matrix by raising it to a power β (β = 6) to amplify the strong connections and penalize the weaker connections [29]. Modules were generated using unsupervised average-linked hierarchical clustering with a cut-off of 0.9. This cut-off was chosen to minimize a large number of modules with very few genes, that is, less than 20 modules containing at least 100 genes. Each module is a hierarchical gene network. Gene significance (GS): defined as GSi = |cor(x_i_, T)|, indicates correlation of a x_i _node expression profile to a phenotypic trait T, a binary trait variable across m samples [29]. In this case, phenotypic trait is lung tissue type - ADC, SCC, SCLC, and NL. Network hubs are defined as highly connected genes within a network, having high intramodular connectivity. Intramodular connectivity is a measure of module eigengene-based connectivity (kME) (or module membership), defined as K_cor,i_^(q) ^= cor(x_i, _E^(q)^), where E^(q) ^is the module eigengene or 1^st ^principal component of module q. Module hubs that have high GS are hubs that are significantly correlated to a phenotypic trait [29], in our case, SCLC phenotype. To filter hubs significantly correlated to SCLC phenotype and identify a SCLC specific hub network (SSHN), we used high values of GS, kME and differential expression (SCLC vs normal lung NL). To classify SCLC from other lung cancer types, unsupervised clustering of the SSHN genes was performed by bootstrapping analysis using pvclust package [69]. Bootstrapping analysis provides confidence values for the stability of each cluster derived by hierarchical clustering, via resampling of the data. Heatmaps were generated using the gplots package [70]. For classification performance estimation, we used nested repeated 5-fold cross-validation procedure [71]. The inner loop of cross-validation was used to determine the best parameters of the classifier (i.e., values of parameters yielding the best classification performance for the validation dataset). The outer loop of cross-validation was used for estimating the classification performance of the model that was built using the previously found best parameters by testing with an *independent set of samples*. To account for variance in performance estimation, we repeated this entire process (nested 5-fold cross-validation) for 10 different splits of the data into 5 cross-validation testing sets and averaged the results. Linear support vector machine is used as the classifier in our analysis, and the error penalty parameter was selected based on the nested cross-validation procedure.

### Pathway analysis

Functional enrichment analysis of the SCLC hub network (SSHN) was performed using Webgestalt [33]. This tool statistically compares the enrichment of SSHN genes with pathways contained in various databases such as Gene Ontology (GO), Kyoto Encyclopedia of Genes and Genomes (KEGG). Functional category enrichment in Webgestalt was tested by the hypergeometric test and multiple comparison corrections were made using Benjamini & Hochberg method [33,72].

### RNAseq data generation and analysis

Tissue samples (20 samples: 10 with SCLC, 5 with SCC, and 5 normal bronchial brushings) were collected from the Vanderbilt University Medical Center and the University of Liverpool Hospital. Research protocols were approved by both institutions' Institutional Review Board. Total RNA was extracted from fresh frozen tumors and bronchial brushings by the RNeasy Kit (Qiagen, CA USA) according to the manufacturer's protocol. Whole transcriptome analysis (RNA-seq) was carried out by next-generation sequencing using Illumina platform in the lab of Vanderbilt Genome Sciences Resource. Next-generation sequencing methodology has been applied to sequence RNA from 20 tissue samples. Due to staged sequencing of samples, two technologies have been utilized: Illumina GAIIX and Illumina Hi-Seq. Sequencing runs from Illumina GAIIX (for 11 samples) were produced with 43bp reads and data was preprocessed using CASAVA 1.7 software. Sequencing runs from Illumina Hi-Seq (for 9 samples) were produced with 51bp reads and data was preprocessed with CASAVA 1.8 software. To make data from two platforms comparable, we have trimmed the last 8bp on each Illumina Hi-Seq read. Using 20 FASTQ data files (after Illumina Pass Filtering) with 43bp reads, we performed alignment using TopHat (v1.4.1), Bowtie (v0.12.7.0), and Samtools (v0.1.18) software. We experimented with two alignment approaches: with two seeds of 21bp and with one seed of 25bp. Since both alignment approaches led to very similar results (in terms of number and percentage of pass filter aligned reads and gene correlations with phenotypes in Fragments Per Kilobase of transcript per Million (FPKM) mapped reads data), we decided to use alignment with one seed of 25bp. Given aligned data, we computed gene expression FPKM (fragments per kilobase of exon per million fragments mapped) values using Cufflinks (v1.3.0) software and performed additional upper quintile normalization of Cufflinks. Using the resulting normalized gene expression dataset, we have assessed whether 287 SSHN genes are associated with SCLC vs. normal OR SCLC vs. SCC brushings by a two-sample t-test at 5% alpha level adjusted for multiple comparisons using the method [61].

### Shotgun Proteomics

Shotgun proteomic analysis was performed from archival formalin fixed paraffin embedded tissues for pools of 5 ADC, 5 SCC, 5 SCLC as well as 5 non-cancerous alveolar lung and 5 bronchial epithelium tissue using our previously published methods [73]. Briefly, following deparaffinization with Sub-X, rehydration with ethanol-water, and protein solubilization in ammonium bicarbonate and trifluoroethanol, proteins were reduced, alkylated and digested overnight with trypsin. Tryptic peptides were separated by isoelectric focusing using ZOOM IPGRunner IEF strips (Invitrogen) with an immobilized pH gradient of 3.5-4.7 [74]. LC-MS/MS analyses were performed on an LTQ-XL mass spectrometer (Thermo Fisher Scientific, San Jose, CA) equipped with an Eksigent nanoLC 1D plus pump and Eksigent autosampler (Dublin, CA) as described previously [73]. MS/MS spectra were processed for protein identifications using a data analysis pipeline described previously [75-77]. False positive peptide-spectrum matches were estimated by reversed database search [5] and held at 5%. Further filtering to require at least one identified spectrum per sample across all analyses maintained a protein false discovery rate (FDR) [72] below 5%. To compare protein expression differences between different histology groups (for example, SCLC vs. Normal), we applied our quasi-likelihood model and analysis software QuasiTel to analyze spectral count data [78]. The quasi-likelihood model, with no restriction on the distribution assumptions, is appropriate for modeling count data with overdispersion and/or underdispersion issue that is frequently observed in spectral count data. Multiple comparison adjusted p values (quasi-FDR) were calculated by incorporating the FDR method described previously [72].

### Tissue microarray immunostaining and analysis

Two TMAs of SCLC specimens were prepared from formalin-fixed paraffin-embedded (FFPE) tissue blocks following previously reported methods [40]. Pathology blocks were retrieved from the archives of the Department of Pathology at Vanderbilt University Medical Center, Nashville VA Medical Center and St-Thomas Hospital in Nashville, Tennessee. They were obtained between 1996 and 2008 from 85 patients who had surgery or bronchoscopy prior to medical treatment. SCLC diagnosis was confirmed on hematoxylin and eosin-stained sections by an experienced lung cancer pathologist (RE). The study was approved by Institutional Review Boards at each medical center. The Syk/Fyn IHC was examined in two to five spots for each TMA. The intensity of staining was scored as 0-no staining, 1-weak, 2-moderate, and 3-strong and the percentage of area stained was also measured. The IHC score was determined by multiplying intensity score to the percentage area stained. The highest score among the spots was used for the unsupervised clustering analysis of Syk/Fyn expression. Tumor images were captured by brighfield microscopy using the Leica SCN400 system (Leica Biosystems^®^) at 20X magnification.

### Western blot

All cell lines were plated for 2 days in complete medium to achieve equilibrium in signaling states. Lysates were prepared by spinning cells down at 4°C, aspirating the media, and adding M-PER lysis buffer (Pierce^®^) containing 1X phosphatase inhibitors 2 and 3 and protease inhibitor (Sigma-Aldrich^®^). Lysates were incubated for five minutes at room temperature, vortexed for 30secs and centrifuged at 15000 rpm for 15mins (at 4°C). The protein concentration was quantified using BCA assay (Pierce^®^) and 30ug of protein was loaded onto 8% Bis-tris gels (Bio-Rad^®^). Blots were imaged using chemiluminescence or Odyssey. The band intensities were quantified using ImageJ and plotted in R (http://www.r-project.org). For siRNA experiments, 400,000 cells were transfected using Dharmafect 4 transfection reagent and siRNA (Dharmacon^®^) in 6-well plates. Cells were incubated for either 3 or 7 days followed by lysate preparation and western blotting process as detailed above.

### Viability assay

10000 cells (of H69 or H146) were plated in 100ul of complete medium (RPMI 1640 containing 10% Fetal bovine serum) in a 96-well plate with Dharmafect 4 and siRNA mixture. The reagent dilutions and transfection procedures were performed as per the manufacturer's protocol. Cells were incubated at 37°C until each timepoint. At each timepoint, cells were transferred to a BD Falcon 96-well black clear bottom imaging plate and live-dead viability dyes (calcein - live cells; ethidium homodimer - dead cells) and hoescht 33342 for total nuclei (Invitrogen^®^) were added in complete medium. The cells were incubated with the dyes for 15mins at 37°C followed by imaging using the Cellavista high-throughput imaging microscope (SynenTec, Elmshorn, Germany). The Roche cell viability protocol was used to image and quantify the cells in 3 colors as per manufacturer's instructions. The output generated from this algorithm included total cell number, viable cell count, percent live/dead cells, etc. The data plotting and statistics were done using R [66]. The viability growth curves statistics were generated using a linear regression growth model [79]. Multiple comparison of treatments were derived using ANOVA and Tukey's method [79,80]. The *p*-values for percent dead at day 5 were generated using a paired t-test, pairing across, N = *4*, experimental replicates.

## Competing interests

The authors declare that they have no competing interests.

## Authors' contributions

AU conceived, designed the study, performed the experiments, analyzed data and wrote the article. MDH, JEC and YZ performed experiments. ZT, ZL, ML, HC, AS contributed to statistical input and data analysis for RNAseq, shotgun proteomics and TMA. RE contributed to TMA distribution and provided pathology scoring of the patient TMA staining. YS and DCL contributed to valuable insight into the overall design of the study and data analysis. JF contributed with clinical samples and discussions. VQ, LE and PPM contributed to conception and design of the study and revising and writing of the article. All authors read and approved the manuscript.

## Supplementary Material

Additional file 1 **This file includes the following supplementary figures 1-5. Figure S1: Absence of modules/clusters in a control WGCNA analysis of a simulated random dataset**. 1000 random datasets were simulated in R to mirror the test dataset GSE6044 (8500 genes, 33 samples)[9], and was subjected to the exact analysis. (A) A representative dendrogram is shown (each line is a gene). Essentially all genes merged into the grey module, which is reserved by WGCNA to genes not assigned to any module. (B) Shows the number of random simulated datasets from the N = 1000 that detected a certain number of modules. The overall p-value for this simulation analysis is less than 0.001, which is highly significant, indicating that our 13 modules detected in GSE6044 are meaningful and relevant to the biology of these tumors. **Figure S2: SSHN as a reproducible classifier in GSE11969 and in-house Agilent datasets**. Unsupervised clustering heatmap based on SSHN genes (rows) of (A) 163 lung cancer patients (columns) in GSE11969 dataset [30], and (B) our own Agilent microarray dataset containing 23 SCC and 10 SCLC samples. Red and green colors in rows of the heatmap indicate high and low expression respectively. LCC- large cell lung carcinoma, LCNEC- large cell neuroendocrine carcinoma. **Figure S3: mRNA expression of SSHN genes for the top representative canonical pathways from network enrichment analysis**. Functional enrichment analysis was carried out using Webgestalt [33]. Boxplots of mRNA expression of representative SSHN hubs functioning in various pathways (A) Cell cycle checkpoint control and DNA replication; (B) DNA damage response and repair; (C) Wnt and Notch signaling pathways (D) Amino acid metabolism pathways. The outliers are denoted by dots. P-value shows statistical significance by Kruskal-Wallis nonparametric test [81]. **Figure S4: Viability assay measurements using Cellavista high-throughput imaging microscope**. (A) Individual cell populations and segmentation performed by Cellavista Roche viability kit algorithm. The colors denote the different dyes used for measurement of total cell count (blue, Hoescht 33342 - left image), viable cell count (green, calcein AM - center) and dead cell count (red, ethidium homodimer - right). Representative viability assay images of H146 (top panel) and H69 (bottom panel) - (B) No treatment, (C) Scrambled and (D) SYK siRNA. SYK knock-down decreases cellular viability via increased death in both H69 and H146. **Figure S5: Fyn KD has no effect on Fyn and Syk positive SCLC cell lines**. The SCLC cell lines H146 (A-C) and H69 (D-F) were treated with Syk-specific and control siRNA as described in Materials and Methods section. (A, D) The efficiency of Fyn siRNA inhibition was measured by Western blotting on day 3 and 7 post transfection. Band intensity (lower panels) was quantified by densitometry in ImageJ. (B, E) Cell proliferation, measured by cell counts as described in Materials and Methods section, shows that Fyn-siRNA treatment shows no growth inhibition compared to untreated cells and to scrambled siRNA treatment. Asterisks denote overall statistical significance of the slope as compared to control across the siRNA conditions, as follows: <0.0005 '***' 0.001 '**' 0.01 '*' 0.05 '.'. The viability growth curves (from N = 4 experiments) statistics were generated from slopes of a linear regression model. Multiple comparison of treatments were derived using ANOVA and Tukey's method [80]. (C and F) Percentage of dead cells (percent of ethidium homodimer positive cells normalized to total cell counts, see Materials and Methods) in Fyn siRNA treated cells at day 5, compared to controls. Asterisks denote statistical significance measured by paired *t*-test as compared to control across the siRNA conditions, as follows: <0.0005 '***' 0.001 '**' 0.01 '*' 0.05 '.'. Click here for file

Additional file 2 **Modules identified by WGCNA**. This table shows the 13 modules (column modules) identified by WGCNA analysis. Columns titled kME denote the module specific kME values for each gene assigned by WGCNA. The kME denotes the intramodular connectivity of a gene within a particular module. Gene significance or GS for each lung tissue type is indicated in separate columns. Columns T-AD show fold change and T-test statistic values for SCLC versus normal lung comparisons. Click here for file

Additional file 3 **SCLC specific hub network signature (SSHN) gene information**. This table shows expression values for 287 SSHN genes (identified by WGCNA analysis) across various datasets. RNAseq data shows comparisons of differential expression of SCLC versus normal lung and associated statistics such as p-value and false discovery rates (FDR). Shotgun proteomic data denotes the comparison of rate ratios (obtained from Shotgun data, see Materials and methods) of SCLC versus normal bronchiolar epithelium and SCLC versus normal alveolar epithelium. Note that a few hubs from the yellow and black module (high kMEyellow and kMEblack respectively) with high GS.SCLC and T-test statistic were also included in the SSHN. Shown in this table is only kMEblue. kMEblack and kMEyellow are shown in Additional file 2. Click here for file

Additional file 4 **SCLC specific hub network signature (SSHN) gene Gene ontology (GO) enrichment analysis**. This table shows the enriched GO biological processes and genes within the SSHN contained within those categories. This enrichment analysis was performed using Webgestalt [33] as described in Materials and Methods. For each GO biological process, the first row lists the process name, and corresponding GO ID. The second row lists number of reference genes in the category (C), number of genes in the gene set and also in the category (O), expected number in the category (E), Ratio of enrichment (R), p value from hypergeometric test (rawP), and p value adjusted by the multiple test adjustment (adjP). Finally, genes in the pathway are listed. For each gene, the table lists the Gene symbol, and description. Click here for file

Additional file 5 **SCLC specific hub network signature (SSHN) gene KEGG pathway enrichment analysis**. This table shows the enriched Kyoto Encyclopedia of Genes and Genomes (KEGG) canonical pathways and genes within the SSHN contained within those categories. This enrichment analysis was performed using Webgestalt [33] as described in Materials and Methods. For each KEGG pathway, the first row lists the KEGG pathway name, and corresponding KEGG ID. The second row lists number of reference genes in the category (C), number of genes in the gene set and also in the category (O), expected number in the category (E), Ratio of enrichment (R), p value from hypergeometric test (rawP), and p value adjusted by the multiple test adjustment (adjP). Finally, genes in the pathway are listed. For each gene, the table lists the Gene symbol, and description. Click here for file

Additional file 6 **Kinase hubs of SSHN**. This table shows expression values for twenty kinase genes (identified by WGCNA analysis) enriched in SSHN across various datasets. RNAseq data shows comparisons of differential expression of SCLC versus normal lung and associated statistics such as p-value and False discovery rates (FDR). Shotgun proteomic data denotes the comparison of rate ratios (obtained from Shotgun data, see Materials and methods) of SCLC versus normal bronchiolar epithelium and SCLC versus normal alveolar epithelium. Click here for file

## Declarations

The publication fees were covered by Grant 5 U54 CA113007-09 from the National Cancer Institute, Integrative Cancer Biology Program (NCI ICBP), awarded to Dr. Vito Quaranta

This article has been published as part of *BMC Systems Biology *Volume 7 Supplement 5, 2013: Selected articles from the International Conference on Intelligent Biology and Medicine (ICIBM 2013): Systems Biology. The full contents of the supplement are available online at http://www.biomedcentral.com/bmcsystbiol/supplements/7/S5.
