# Supplementary material for: Co-expression network analysis identifies Spleen Tyrosine Kinase (SYK) as a candidate oncogenic driver in a subset of small-cell lung cancer
Source: BMC Syst Biol. 2013 Dec 9;7(Suppl 5):S1. doi: 10.1186/1752-0509-7-S5-S1 (PMC4029366; doi:10.1186/1752-0509-7-S5-S1)
Supplement: Additional file 1 — This file includes the following supplementary figures 1-5. Figure S1: Absence of modules/clusters in a control WGCNA analysis of a simulated random dataset. 1000 random datasets were simulated in R to mirror the test dataset GSE6044 (8500 genes, 33 samples)[9], and was subjected to the exact analysis. (A) A representative dendrogram is shown (each line is a gene). Essentially all genes merged into the grey module, which is reserved by WGCNA to genes not assigned to any module. (B) Shows the number of random simulated datasets from the N = 1000 that detected a certain number of modules. The overall p-value for this simulation analysis is less than 0.001, which is highly significant, indicating that our 13 modules detected in GSE6044 are meaningful and relevant to the biology of these tumors. Figure S2: SSHN as a reproducible classifier in GSE11969 and in-house Agilent datasets. Unsupervised clustering heatmap based on SSHN genes (rows) of (A) 163 lung cancer patients (columns) in GSE11969 dataset [30], and (B) our own Agilent microarray dataset containing 23 SCC and 10 SCLC samples. Red and green colors in rows of the heatmap indicate high and low expression respectively. LCC- large cell lung carcinoma, LCNEC- large cell neuroendocrine carcinoma. Figure S3: mRNA expression of SSHN genes for the top representative canonical pathways from network enrichment analysis. Functional enrichment analysis was carried out using Webgestalt [33]. Boxplots of mRNA expression of representative SSHN hubs functioning in various pathways (A) Cell cycle checkpoint control and DNA replication; (B) DNA damage response and repair; (C) Wnt and Notch signaling pathways (D) Amino acid metabolism pathways. The outliers are denoted by dots. P-value shows statistical significance by Kruskal-Wallis nonparametric test [81]. Figure S4: Viability assay measurements using Cellavista high-throughput imaging microscope. (A) Individual cell populations and segmentation performed by Cellavista Roche vi [file 1752-0509-7-S5-S1-S1.PDF]

Figure S1

A

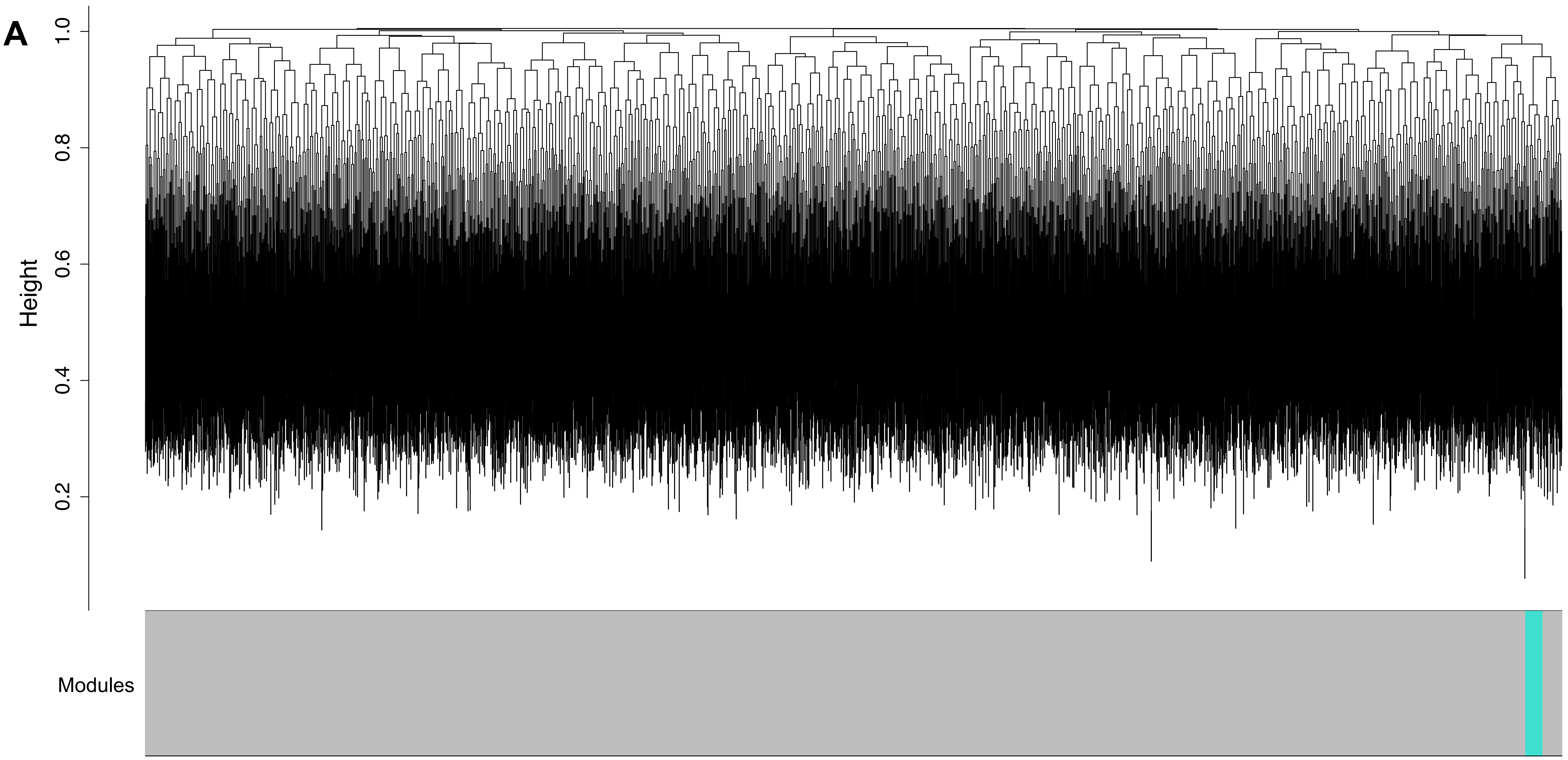

B

Number of clusters/modules	Number of datasets
8	1
7	1
6	6
5	24
4	74
3	167
2	277
1	285
0	165

Figure S2

A

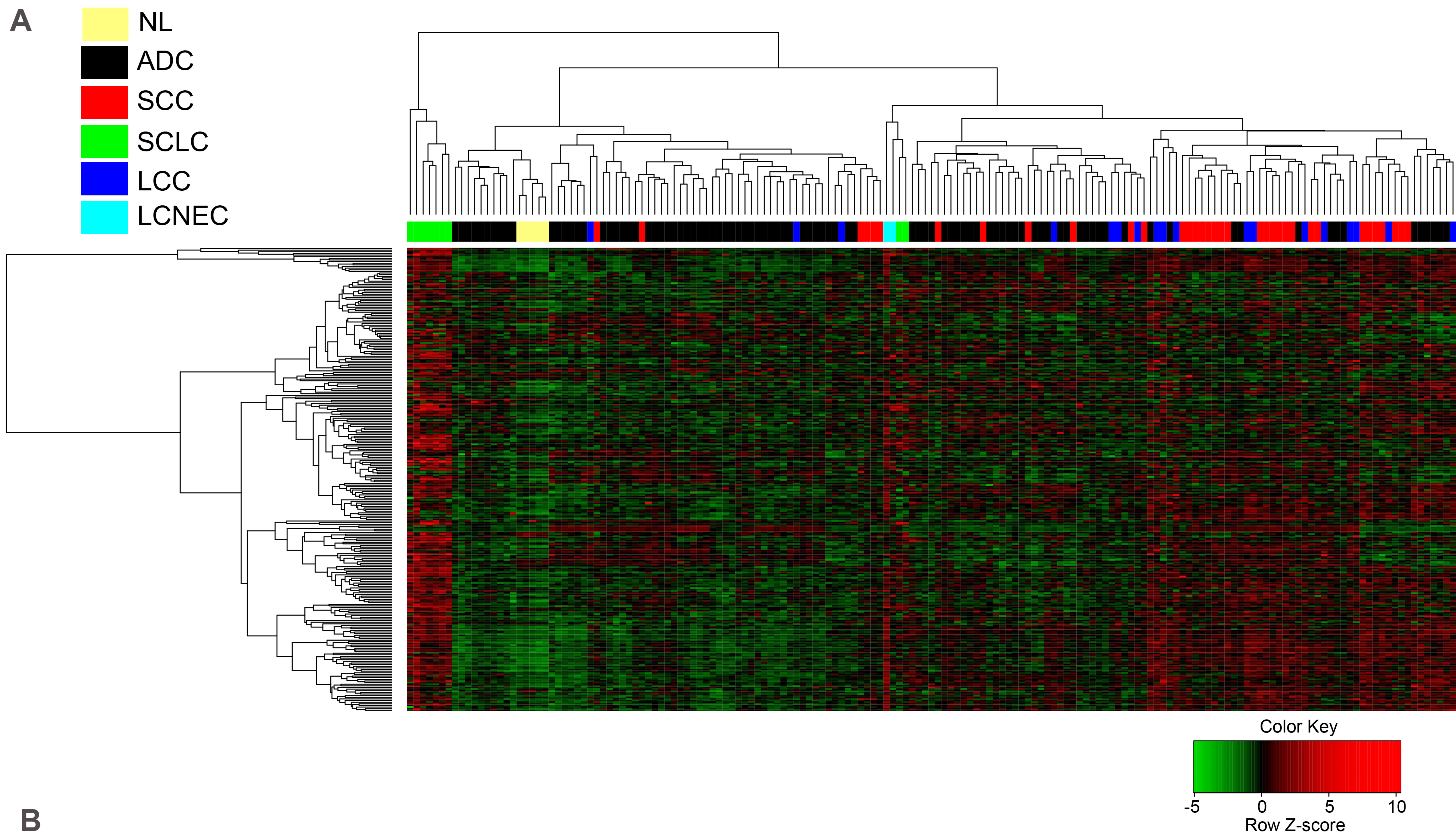
NL
ADC
SCC
SCLC
LCC
LCNEC
Color Key
-5
0
5
10
Row Z-score

B

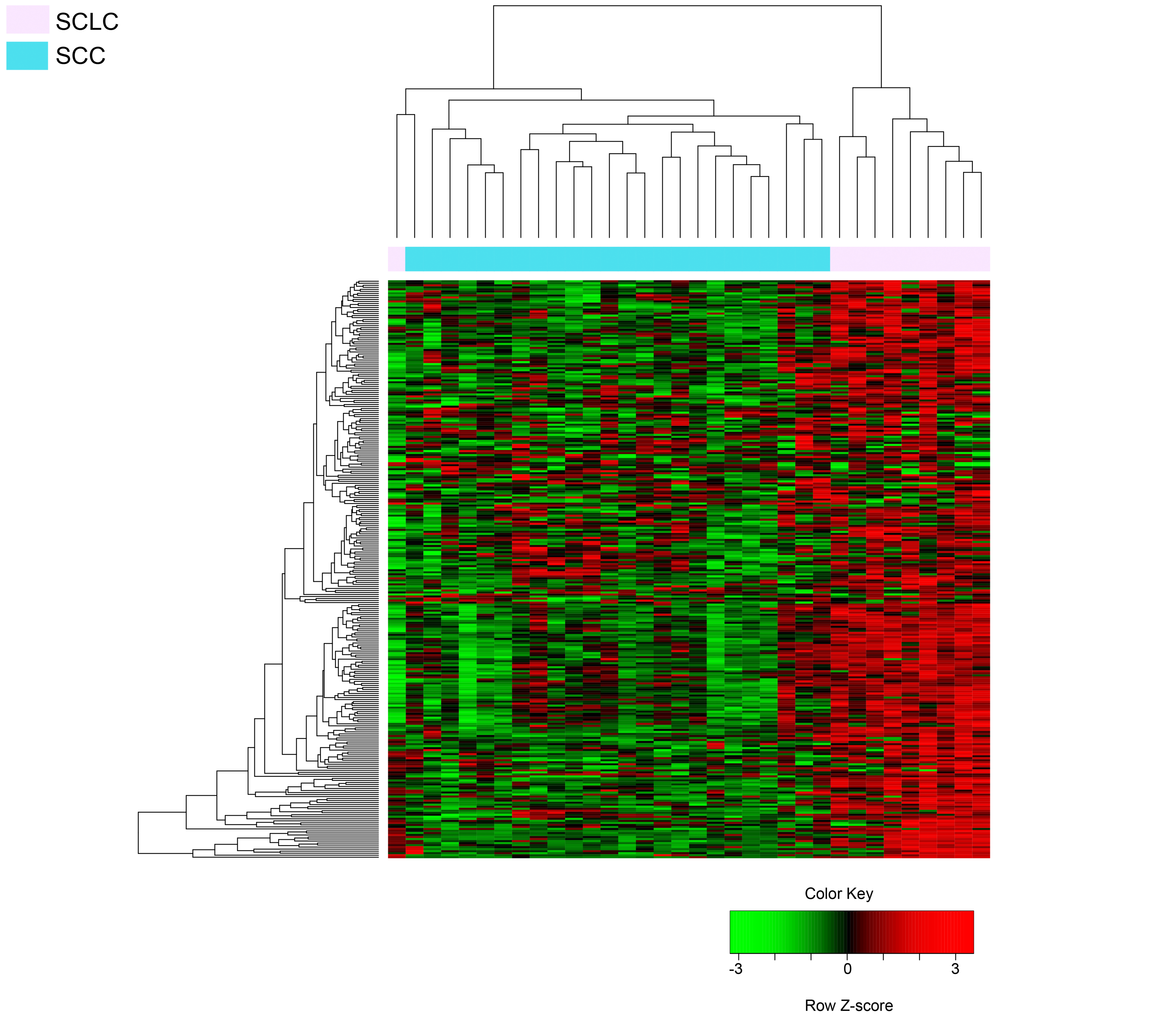
SCLC
SCC
Color Key
-3
0
3
Row Z-score

Figure S3

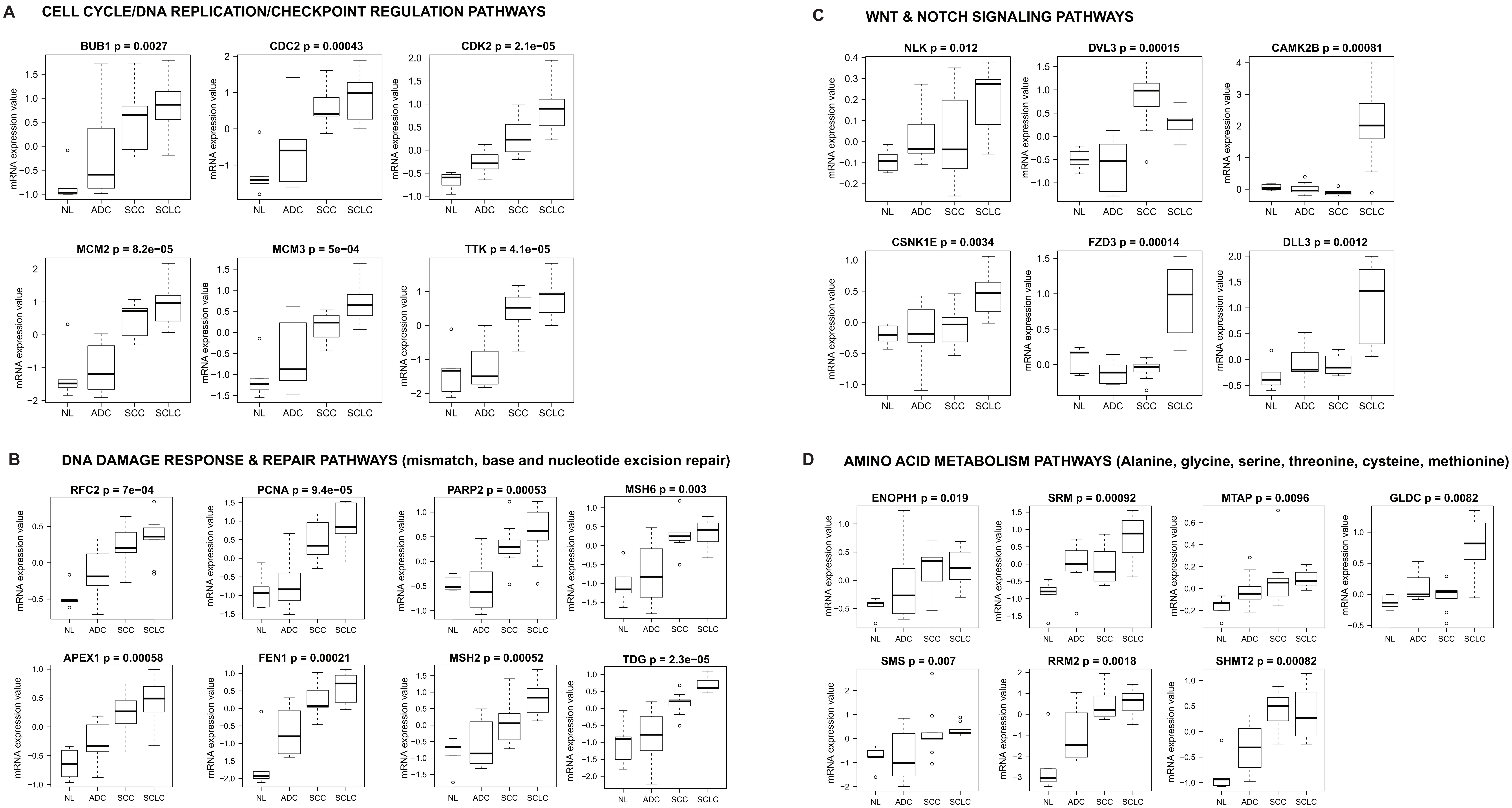

A
CELL CYCLE/DNA REPLICATION/CHECKPOINT REGULATION PATHWAYS
BUB1 p = 0.0027
CDC2 p = 0.00043
CDK2 p = 2.1e−05
MCM2 p = 8.2e−05
MCM3 p = 5e−04
TTK p = 4.1e−05
B
DNA DAMAGE RESPONSE & REPAIR PATHWAYS (mismatch, base and nucleotide excision repair)
RFC2 p = 7e−04
PCNA p = 9.4e−05
PARP2 p = 0.00053
MSH6 p = 0.003
APEX1 p = 0.00058
FEN1 p = 0.00021
MSH2 p = 0.00052
TDG p = 2.3e−05
C
WNT & NOTCH SIGNALING PATHWAYS
NLK p = 0.012
DVL3 p = 0.00015
CAMK2B p = 0.00081
CSNK1E p = 0.0034
FZD3 p = 0.00014
DLL3 p = 0.0012
D
AMINO ACID METABOLISM PATHWAYS (Alanine, glycine, serine, threonine, cysteine, methionine)
ENOPH1 p = 0.019
SRM p = 0.00092
MTAP p = 0.0096
GLDC p = 0.0082
SMS p = 0.007
RRM2 p = 0.0018
SHMT2 p = 0.00082
mRNA expression value
NL
ADC
SCC
SCLC

Figure S4

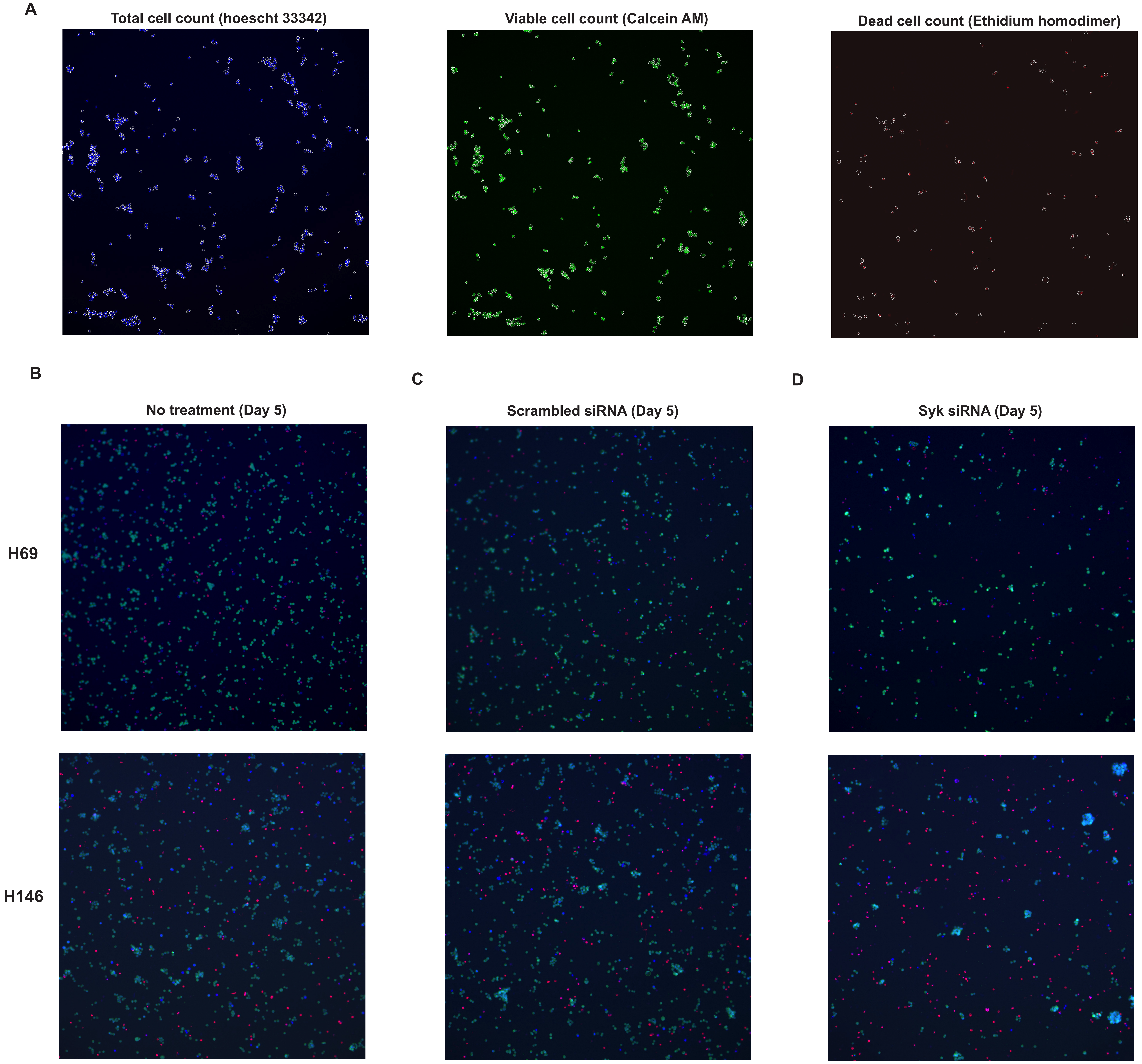
A
Total cell count (hoescht 33342)
Viable cell count (Calcein AM)
Dead cell count (Ethidium homodimer)
B
C
D
No treatment (Day 5)
Scrambled siRNA (Day 5)
Syk siRNA (Day 5)
H69
H146

Figure S5

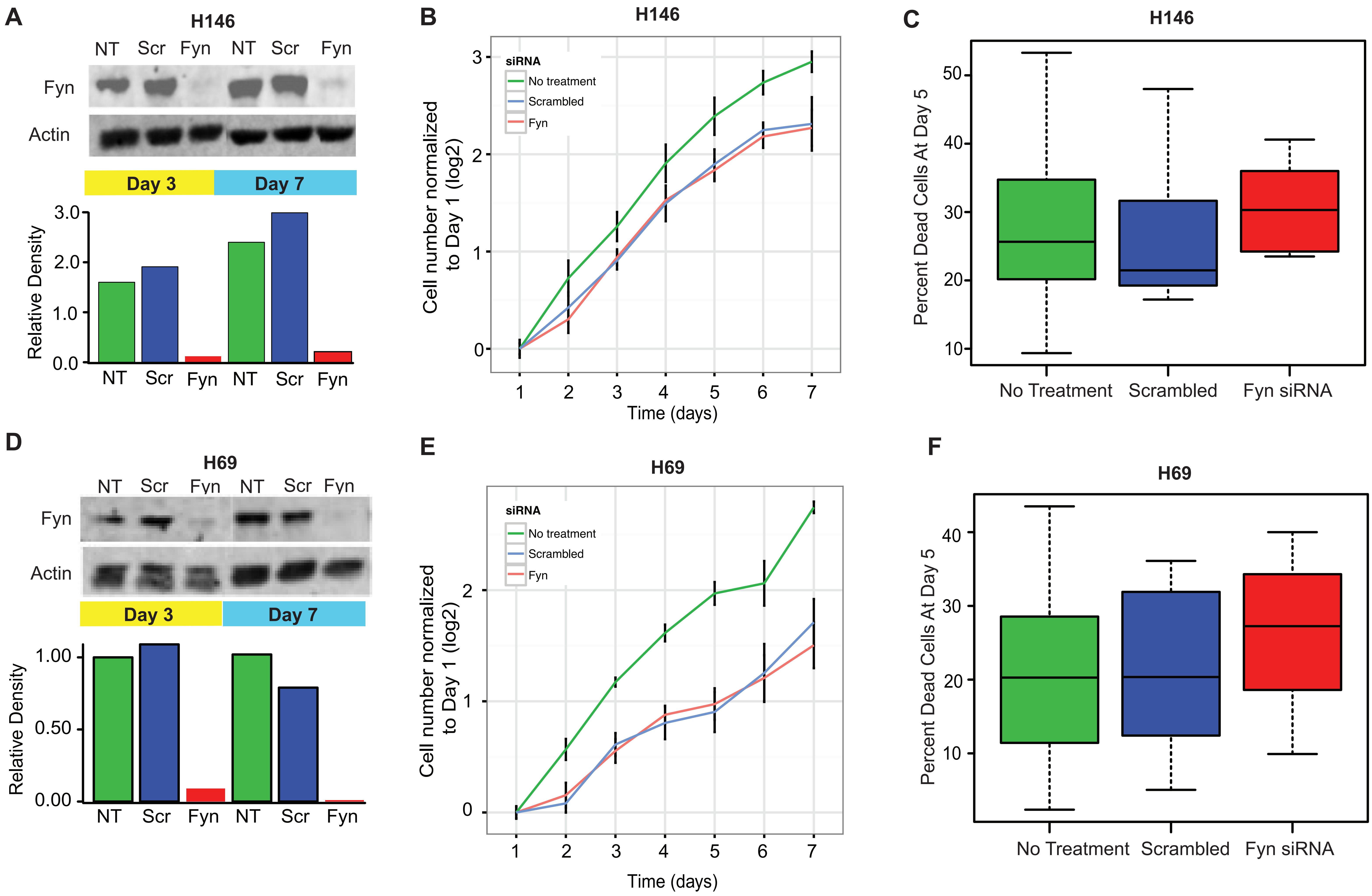

A
H146
NT Scr Fyn NT Scr Fyn
Fyn
Actin
Day 3
Day 7
Relative Density
3.0
2.0
1.0
0.0
NT Scr Fyn NT Scr Fyn
B
H146
siRNA
No treatment
Scrambled
Fyn
Cell number normalized to Day 1 (log2)
Time (days)
C
H146
Percent Dead Cells At Day 5
No Treatment
Scrambled
Fyn siRNA
D
H69
NT Scr Fyn NT Scr Fyn
Fyn
Actin
Day 3
Day 7
Relative Density
1.00
0.50
0.00
NT Scr Fyn NT Scr Fyn
E
H69
siRNA
No treatment
Scrambled
Fyn
Cell number normalized to Day 1 (log2)
Time (days)
F
H69
Percent Dead Cells At Day 5
No Treatment
Scrambled
Fyn siRNA
